# The NLRP3 inflammasome in macrophages is stimulated by cell‐free hemoglobin

**DOI:** 10.14814/phy2.14589

**Published:** 2020-10-31

**Authors:** Ciara M. Shaver, Stuart R. Landstreet, Sangamithra Pugazenthi, Fiona Scott, Nathan Putz, Lorraine B. Ware, Julie A. Bastarache

**Affiliations:** ^1^ Division of Allergy, Pulmonary, and Critical Care Medicine Department of Medicine Vanderbilt University Medical Center Nashville TN USA; ^2^ Vanderbilt University Nashville TN USA; ^3^ Department of Pathology, Microbiology, and Immunology Vanderbilt University Medical Center Nashville TN USA; ^4^ Department of Cell and Developmental Biology Vanderbilt University Nashville TN USA

**Keywords:** acute lung injury, ARDS, cell‐free hemoglobin, inflammasome, NLRP3

## Abstract

Cell‐free hemoglobin (CFH) is associated with severe lung injury in human patients and is sufficient to induce airspace inflammation and alveolar–capillary barrier dysfunction in an experimental model of acute lung injury. The mechanisms through which this occurs are unknown. One key pathway which regulates inflammation during acute lung injury is the NLRP3 inflammasome. Because CFH can act as a damage‐associated molecular pattern, we hypothesized that CFH may activate the NLRP3 inflammasome during acute lung injury. Primary mouse alveolar macrophages and cultured murine macrophages exposed to CFH (0–1 mg/ml) for 24 hr demonstrated robust upregulation of the NLRP3 inflammasome components NLRP3, caspase‐1, and caspase‐11. Maximal induction of the NLRP3 inflammasome by CFH required TLR4. Compared to wild‐type controls, mice lacking NLRP3 developed less airspace inflammation (2.7 × 10^5^ cells/ml in bronchoalveolar lavage fluid versus. 1.1 × 10^5^/ml, *p* = .006) after exposure to intratracheal CFH. Together, these data demonstrate that CFH can stimulate the NLRP3 inflammasome in macrophages and that this pathway may be important in the pathogenesis of CFH‐induced acute lung injury.

## Graphical abstract

Cell‐free hemoglobin is associated with severe lung injury in human patients and is sufficient to induce airspace inflammation and alveolar–capillary barrier dysfunction in an experimental model of acute lung injury. Here, we investigate the role of the NLRP3 inflammasome in lung injury caused by cell‐free hemoglobin using in vitro and in vivo model systems. Our results show that cell‐free hemoglobin increases NLRP3 expression in alveolar macrophages and that mice lacking NLRP3 have reduced inflammation in response to cell‐free hemoglobin, suggesting that NLRP3 has an important role in acute lung injury caused by cell‐free hemoglobin.

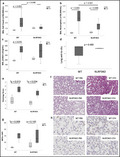

## 1 INTRODUCTION

Acute respiratory distress syndrome (ARDS) is a syndrome of severe lung injury that occurs in the setting of many different illnesses including pneumonia, sepsis, trauma, or after blood transfusion (Shaver & Bastarache, 2014; Ware & Matthay, 2000). ARDS continues to have a mortality rate of approximately 30% despite critical care interventions and there are no targeted therapies available to interrupt the pathogenesis of this disease. Our group previously identified that there are elevated levels of cell‐free hemoglobin (CFH) in the airspace of patients with ARDS (Bastarache et al., 2012) and we proposed that CFH may be an independent contributor to the pathogenesis of acute lung injury. To study this, we developed a model of CFH‐induced lung injury and demonstrated that instillation of purified CFH into the airspace by intra‐tracheal injection is sufficient to cause acute lung injury in mice (Shaver, Upchurch, et al., 2016). This CFH‐induced lung injury mimics the features of human ARDS, including neutrophilic airspace inflammation and disruption of the alveolar–capillary barrier (Shaver, Upchurch, et al., 2016). The mechanisms through which CFH contributes to acute lung injury are not well understood. Several in vitro and in vivo studies have shown that CFH can injure the lung epithelium (Chintagari, Jana, & Alayash, 2016; Mumby, Ramakrishnan, Evans, Griffiths, & Quinlan, 2014; Shaver, Upchurch, et al., 2016) or endothelium (Kuck et al., 2018; Meegan et al., 2020; Shaver et al., 2018). However, much less is known about the underlying mechanisms through which CFH causes airspace inflammation.

When CFH is released into the extracellular environment, it is prone to oxidation and can act as a damage‐associated molecular pattern (DAMP) (Lee & Ding, 2013; Mendonca, Silveira, & Conran, 2016). DAMPs can trigger inflammatory responses by engaging a wide variety of surface receptors which activate NF‐κB and other signaling pathways. One common pathway stimulated by DAMPs is the NLRP3 inflammasome, a complex of proteins that, after priming and activation, regulates inflammatory responses particularly through release of IL‐1β (Lee, Suh, Ryter, and Choi, 2016) More specifically, in the priming step, DAMPs bind to TLR4 or other surface receptors and induce transcription and translation of NLRP3 complex proteins. Then, after a second signal such as reactive oxygen species production or ATP release is detected, NLRP3 assembles with the adaptor protein Asc and activates either caspase‐1 (canonical pathway) or caspase‐11 (non‐canonical pathway). This results in activation of caspase‐1 or caspase‐11, followed by cleavage of pro‐IL‐1β to IL‐1β, which is released from the cell. Previous work has shown that the NLRP3 inflammasome pathway is activated by lipopolysaccharide, microbial products, extracellular DNA, cigarette smoke, and other substances during acute and chronic lung injury (Hosseinian, Cho, Lockey, & Kolliputi, 2015; Lee et al., 2016; Pinkerton et al., 2017) including pneumonia, ARDS, asthma, chronic obstructive pulmonary disease (COPD), and pulmonary fibrosis. Whether the NLRP3 inflammasome is important in CFH‐induced acute lung injury remains unknown.

Because CFH can act as a DAMP and because IL‐1β was expressed in the airspace of mice exposed to intra‐tracheal CFH (Shaver, Upchurch, et al., 2016), we hypothesized that the NLRP3 inflammasome is a key mediator of CFH‐induced lung injury. To test this, we performed a series of experiments using a combination of cultured macrophages, primary alveolar macrophages, and transgenic knockout mice to understand whether CFH can upregulate the NLRP3 inflammasome.

## 2 MATERIALS AND METHODS

### 2.1 Mouse strains

Male and female NLRP3KO mice on a C57Bl/6 background, (Kovarova et al., 2012) aged 6–12 weeks, were raised in our animal facility and WT C57Bl/6 mice were purchased from Jackson Laboratories (Bar Harbor, ME). Alveolar macrophages from global TLR4 knockout mice on a C57Bl/6 background (Jackson Labs, strain 007227) were used for evaluation of NLRP3 dependence on TLR4. All animal studies were approved by the Vanderbilt Institutional Animal Care and Use Committee and performed in accordance with institutional guidelines.

### 2.2 Measurement of NLRP3 inflammasome induction

MH‐S alveolar macrophage‐like cells were grown in RPMI‐1640 with 10% fetal bovine serum, 0.5 mM 2‐mercaptoethanol, and 5% penicillin/streptomycin. Primary alveolar macrophages were collected from WT mice by serial bronchoalveolar lavage (BAL), rested for 1 hr to allow for selection of adherent macrophages, enumerated, and aliquoted into tissue culture dishes for experiments. More than 90% of primary cells extracted were alveolar macrophages. Cells were incubated with endotoxin‐free cell‐free hemoglobin (CFH), (Cell Sciences, Canton, MA) (1 mg/ml) for 24 hr. RNA was extracted and cDNA prepared using SuperScript VILO cDNA Synthesis kit (ThermoFisher, Waltham, MA) and then quantitative RT‐PCR of NLRP3, Asc, caspase‐1, and caspase‐11 was performed using primers from ThermoFisher. Protein levels of NLRP3 (ab214185) and caspase‐1 (ab138483) were measured by Western blot of cell lysates prepared in RIPA buffer, using antibodies from Abcam (Cambridge, MA), and were normalized to actin (A2066,Sigma‐Aldrich, St. Louis, MO).

### 2.3 Animal model of cell‐free hemoglobin‐induced acute lung injury

Mice were anesthetized with isoflurane and CFH (100 μg/mouse) was instilled by direct intra‐tracheal injection, as previously described (Shaver, Upchurch, et al., 2016). This dose was chosen to approximate the levels of CFH present in the airspace of patients with ARDS. After 2 hr or 24 hr, mice were euthanized. Bronchoalveolar lavage (BAL) was performed and lungs flash frozen and stored at −80°C until analysis. Inflammatory cells were manually counted in BAL and differentials determined after DiffQuick staining as previously described (Shaver, Grove, et al., 2016; Shaver et al., 2015; Shaver, Upchurch, et al., 2016). BAL cytokines were measured by multiplex assay (MesoScale Discovery, Gaithersburg, MD) and total protein by BCA assay. Wet‐to‐dry lung weight ratios were calculated as previously described (Ma et al., 2015). For histologic analysis, lungs were perfused with 4% paraformaldehyde, embedded in paraffin, and sections stained with hematoxylin and eosin. Immunohistochemistry for myeloperoxidase was performed by the Vanderbilt Tissue Processing Shared Resource. Histology was quantified by manual assessment of 5 non‐overlapping high‐powered fields (40×) for septal thickening, inflammation, edema, and hemorrhage on a scale of 0–4 for each criterion with the sum histologic score calculated as previously described (Bastarache et al., 2012; Frank et al., 2002). Myeloperoxidase was manually quantified by number of positive cells per 20× high‐powered field. All quantification was performed without knowledge of the animal genotypes or treatment groups.

### 2.4 Cell death assessment

Inflammasome activation can induce a form of programmed cell death that leads to cell lysis and release of intracellular contents. Release of lactate dehydrogenase (LDH) was measured in supernatants of MH‐S cells exposed to CFH or in BAL fluid from mice given IT CFH using the LDH‐Glo Cytotoxicity Assay (Promega, Madison, WI).

### 2.5 Statistical analysis

Continuous variables were analyzed among four groups using Kruskal–Wallis comparisons with post hoc Dunn's pairwise comparisons and between two groups using Mann–Whitney *U* comparison or Student's *t* testing, based on whether the data were normally distributed. Analysis was performed using SPSS version 26 (IBM, Armonck, NY).

## 3 RESULTS

### 3.1 Cell‐free hemoglobin induces NLRP3 inflammasome upregulation

**FIGURE 1:**
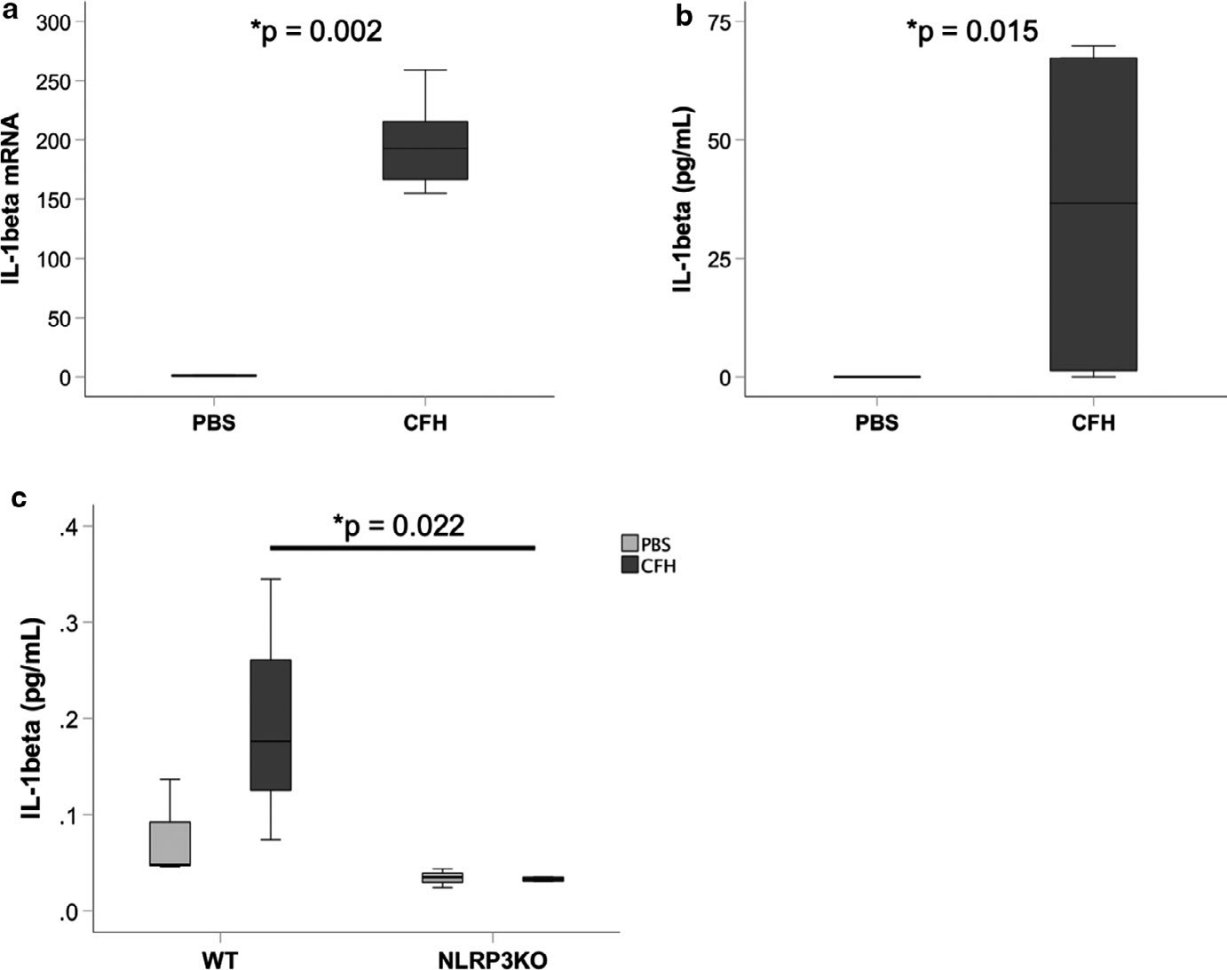
Cell‐free hemoglobin increases expression of IL‐1β in macrophages. Cultured alveolar‐like MH‐S cells were incubated with cell‐free hemoglobin (CFH, 1 mg/ml) or PBS control for 24 hr and IL‐1β expression was measured by (a) RT‐PCR, normalized to mean expression in PBS cells and (b) ELISA. (c) Primary alveolar macrophages from wild‐type (WT) or NLRP3KO mice were incubated with CFH for 24 hr ex vivo and IL‐1β expression measured by MSD. *n* = 6 per group for MH‐S cells and *n* = 3 per treatment condition for primary alveolar macrophages

**FIGURE 2:**
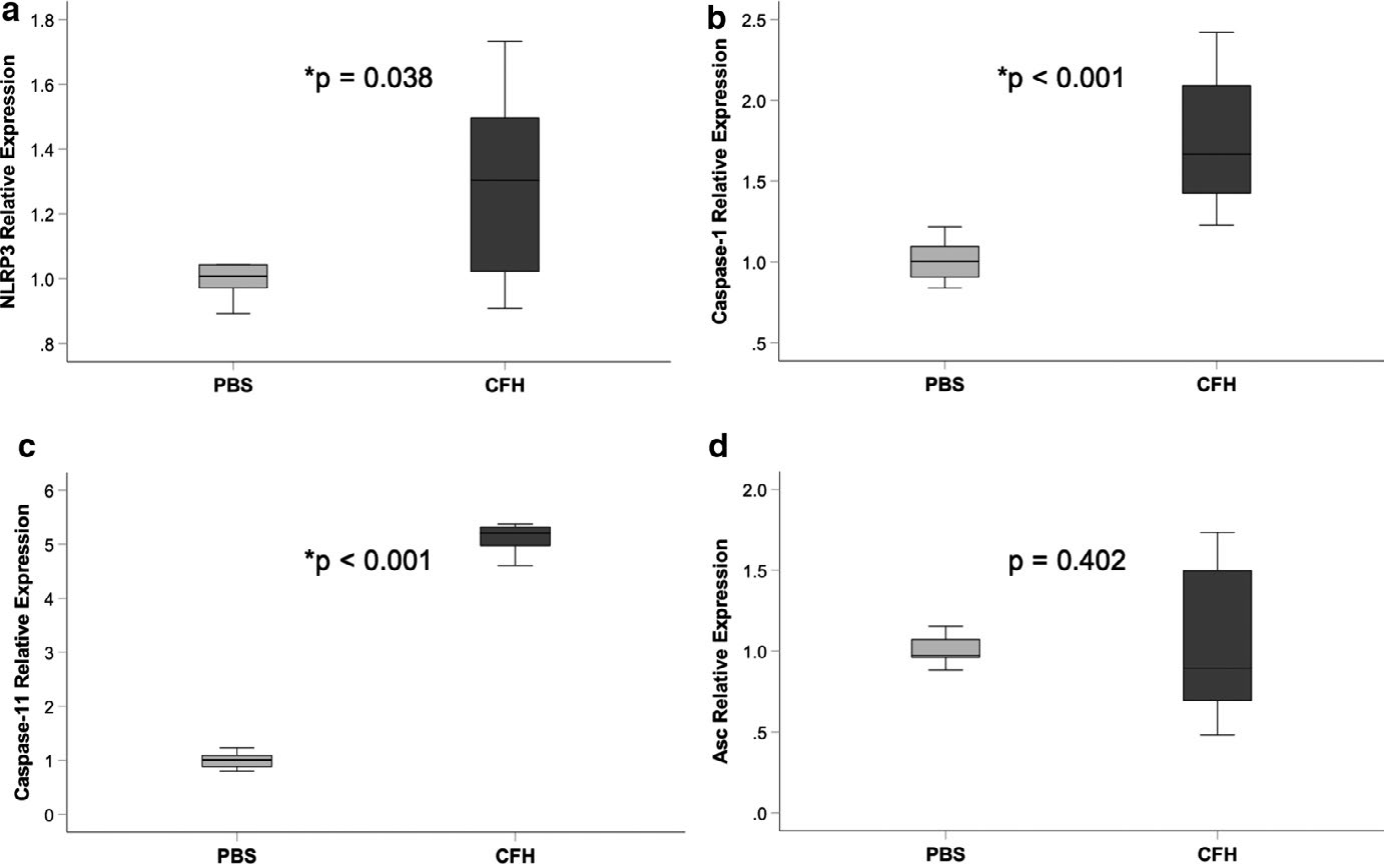
Cell‐free hemoglobin induces NLRP3 inflammasome upregulation in cultured macrophages. Cultured MH‐S macrophages were exposed to CFH (1 mg/ml) or PBS control for 24 hr and upregulation of NLRP3 inflammasome component genes were measured by quantitative RT‐PCR. CFH increased expression of NLRP3 (a), caspase‐1 (b), and caspase‐11 (c), but had no effect on expression of Asc (d). *n* = 9 per group

**FIGURE 3:**
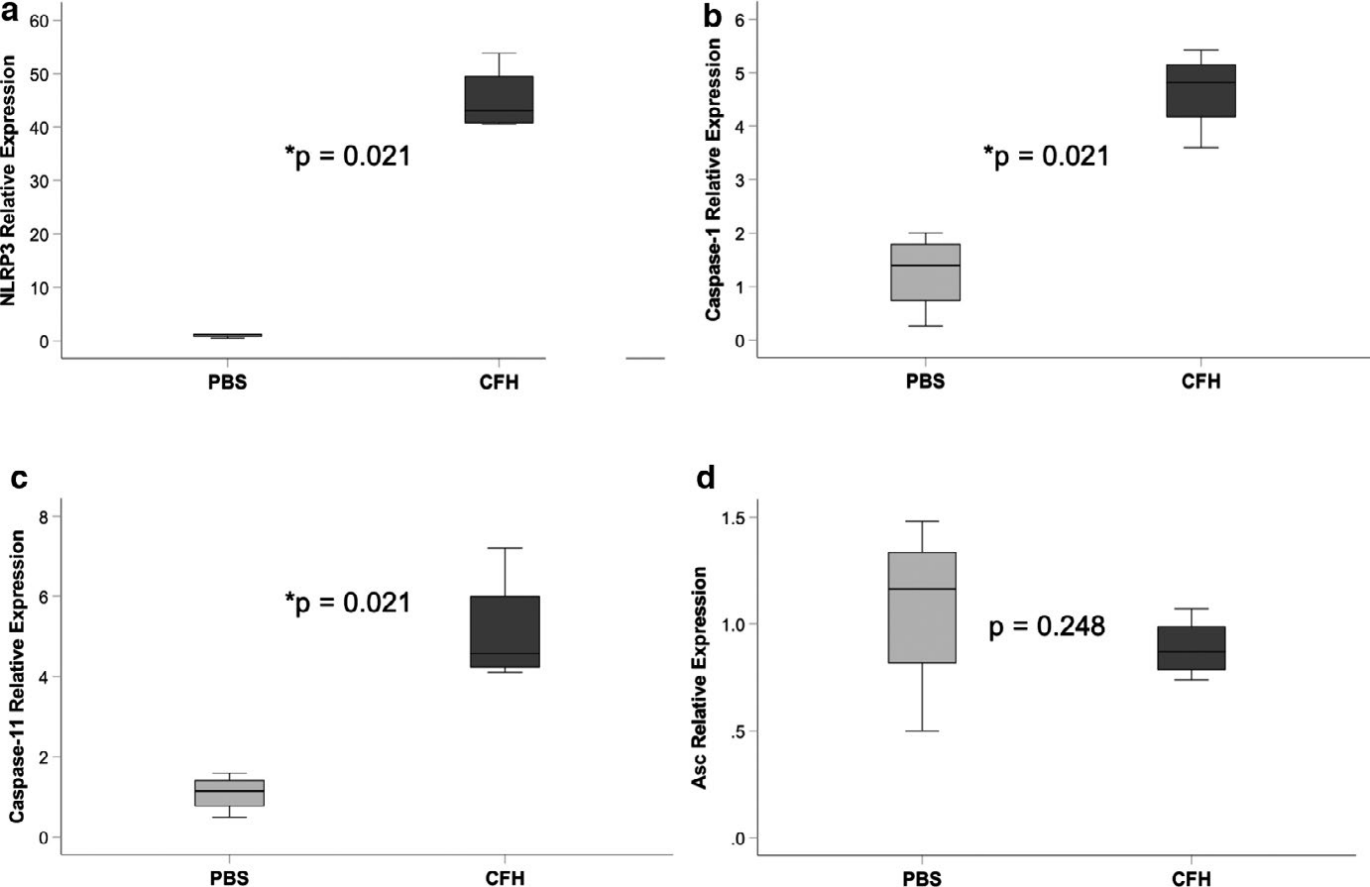
Cell‐free hemoglobin induces NLRP3 inflammasome upregulation in alveolar macrophages. Primary alveolar macrophages from wild‐type mice were exposed to cell‐free hemoglobin (1 mg/ml) or PBS control for 24 hr and upregulation of NLRP3 inflammasome component genes were measured by quantitative RT‐PCR. CFH increased expression of NLRP3 (a), caspase‐1 (b), and caspase‐11 (c), but had no effect on expression of Asc (d). *n* = 4 per group

Our previous study demonstrated that IL‐1β was highly expressed in the airspace of mice exposed to intra‐tracheal CFH (Shaver, Upchurch, et al., 2016). Because IL‐β is the primary cytokine released after NLRP3 activation, we tested whether CFH was sufficient to induce expression of IL‐1β. Cultured MH‐S alveolar‐like macrophages exposed to CFH for 24 hr had increased expression of IL‐1β at the level of mRNA and protein (Figure 1a and b). Primary alveolar macrophages exposed to CFH (1 mg/ml) ex vivo for 24 hr demonstrated increased NLRP3‐dependent production of IL‐1β (Figure 1c). Next, we tested whether CFH was sufficient to induce expression of the NLRP3 inflammasome. NLRP3, caspase‐1, and caspase‐11 mRNA expression increased after CFH exposure, but CFH did not change expression of the adapter protein Asc (Figure 2a–d). To confirm this finding, we also tested whether primary mouse alveolar macrophages had CFH‐dependent upregulation of the NLRP3 inflammasome components. Similar to MH‐S macrophages, primary alveolar macrophages increased expression of NLRP3, caspase‐1, and caspase‐11 in response to CFH (Figure 3a–d).

### 3.2 Cell‐free hemoglobin increases production of NLRP3 and caspase‐1

**FIGURE 4:**
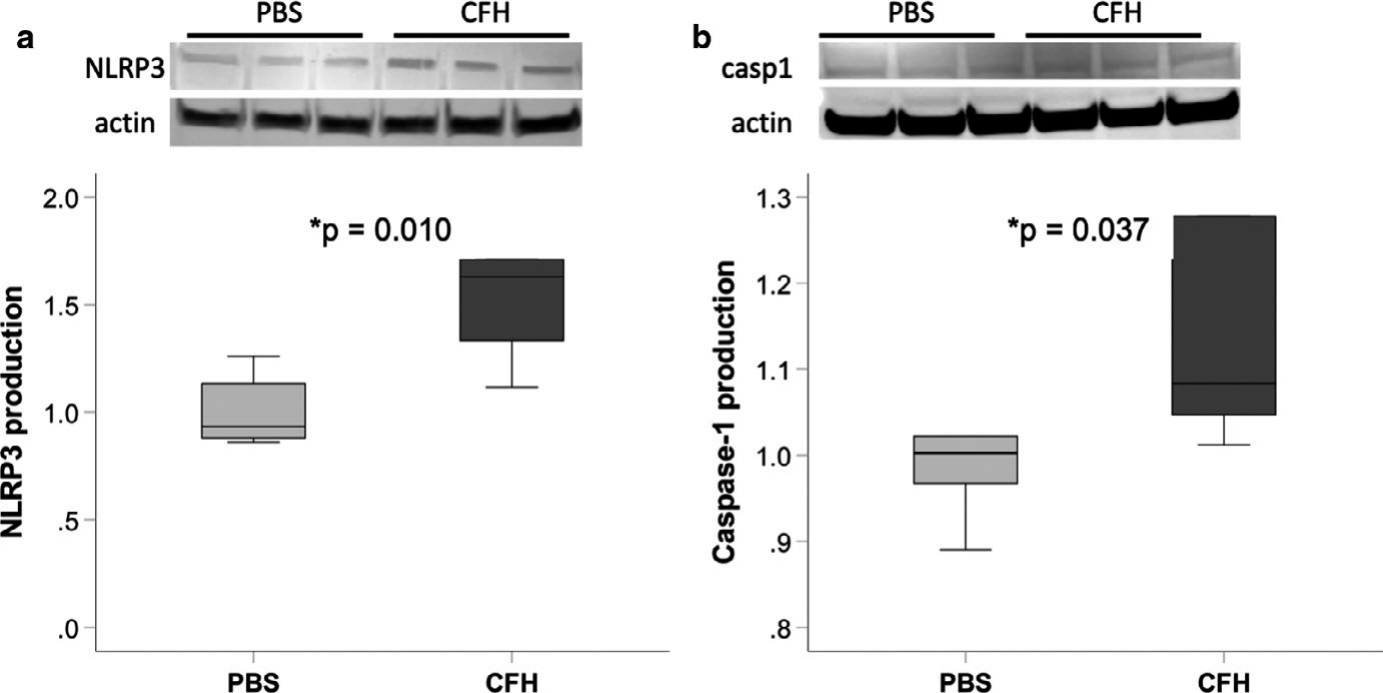
Cell‐free hemoglobin increases protein expression of NLRP3 inflammasome components. Cultured MH‐S macrophages were exposed to CFH (1 mg/ml) for 24 hr. Cell lysates were prepared and equal protein amounts loaded for Western blotting. Quantification of NLRP3 and caspase‐1 was performed and was normalized for actin staining in each lane. *n* = 6 per group

Next, we tested whether exposure to CFH increased protein levels of NLRP3 inflammasome components. Cultured macrophages were incubated with CFH in vitro and cell lysates collected for Western blot analysis. This revealed that exposure to CFH significantly increased expression of both NLRP3 and caspase‐1 (Figure 4a–b). Similar results were obtained from MH‐S macrophages (data not shown).

### 3.3 NLRP3 induction by cell‐free hemoglobin requires TLR4

**FIGURE 5:**
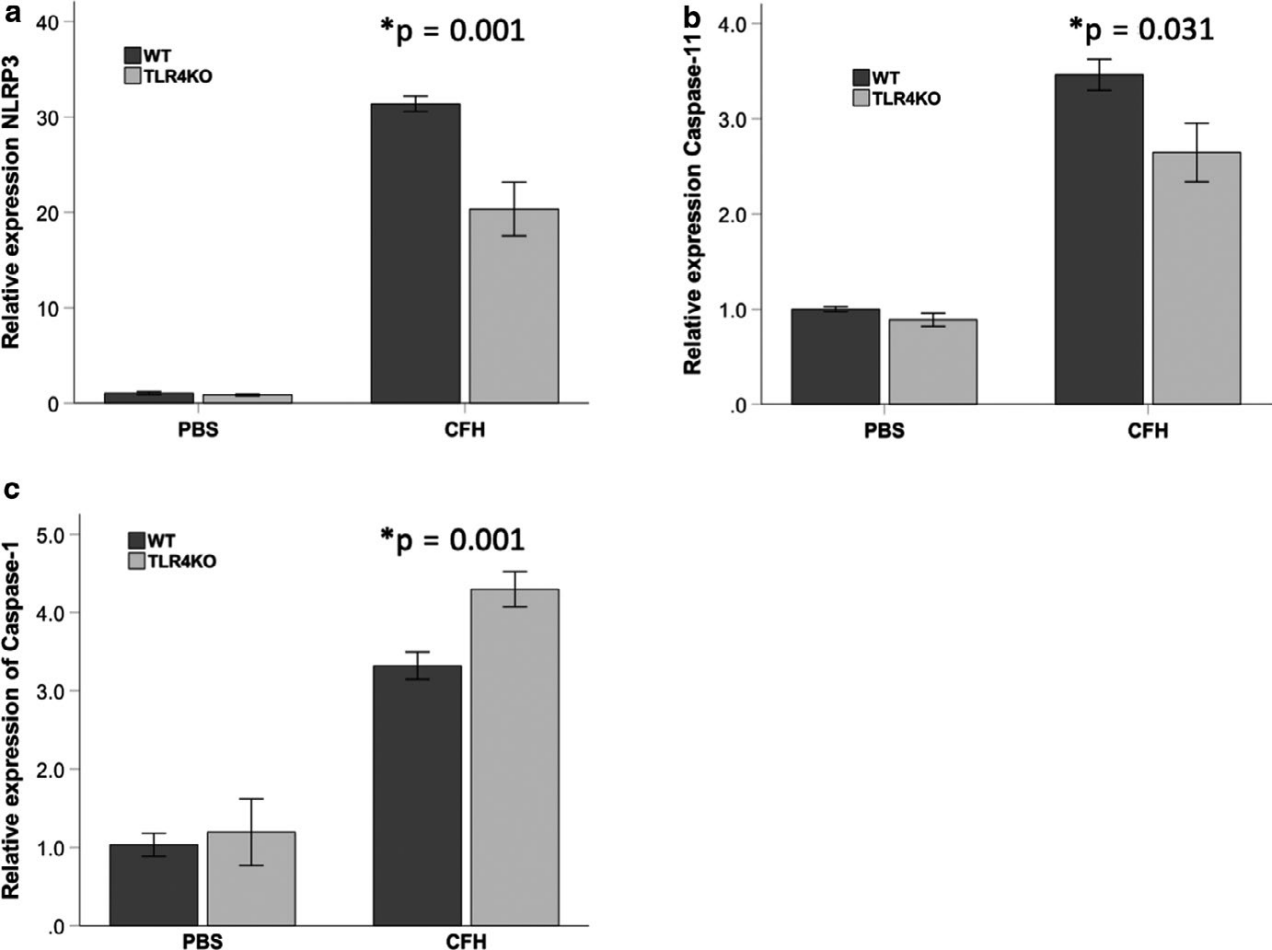
NLRP3 induction by cell‐free hemoglobin requires TLR4. Primary alveolar macrophages from mice lacking TLR4 (TLR4KO) or WT controls were exposed to cell‐free hemoglobin (1 mg/ml) or PBS control for 24 hr. In the absence of TLR4, there was significantly less upregulation of mRNA for (a) NLRP3 and (b) caspase‐11, but not (c) caspase‐1 as measured by RT‐PCR. *n* = 4 per group

TLR4 is one of the surface receptors that can be engaged to induce NLRP3. To determine whether TLR4 was necessary for NLRP3 expression in response to CFH, we tested the impact of genetic TLR4 deletion on upregulation of NLRP3 components. WT or TLR4KO primary alveolar macrophages were exposed to CFH ex vivo and NLRP3 induction was measured. TLR4 was required for CFH‐dependent induction of NLRP3 and caspase‐11, but not caspase‐1 (Figure 5a–c).

### 3.4 NLRP3 inflammasome is required for maximal airspace inflammation in response to cell‐free hemoglobin

**FIGURE 6:**
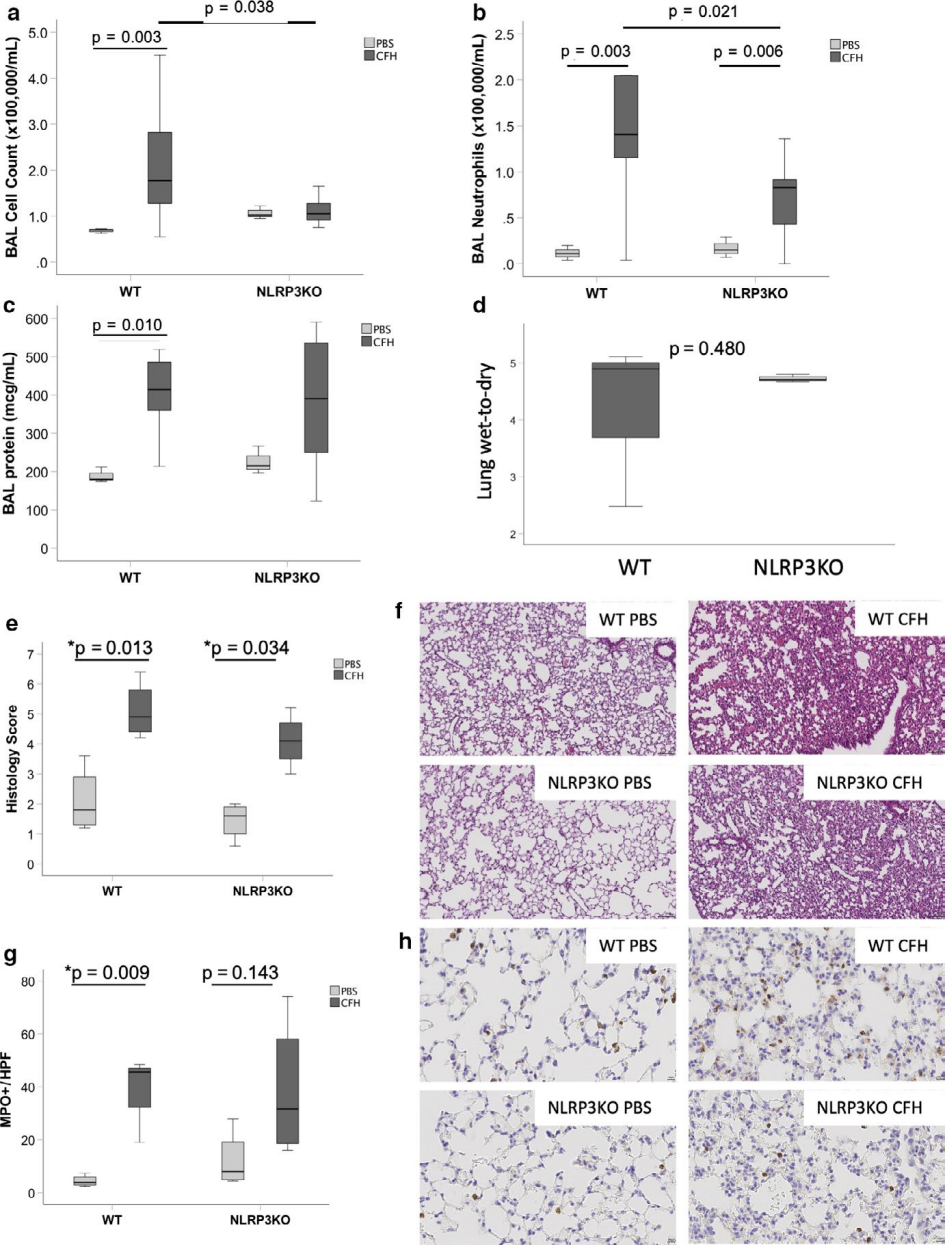
NLRP3 inflammasome is required for maximal airspace inflammation in response to cell‐free hemoglobin. NLRP3KO mice or WT controls were exposed to intra‐tracheal CFH (100 μg) or PBS control. After 24 hr, mice lacking NLRP3 had (a) reduced BAL inflammation with (b) reduced neutrophil influx. There were no significant differences between WT and NLRP3KO CFH mice in bronchoalveolar lavage total protein (c) or in lung wet‐to‐dry weight ratios (d), *n* = 3–4 per group. Histologic analysis by hematoxylin and eosin quantification and staining is shown in Panels E‐F for each group. Quantification of histologic lung injury (20×) and myeloperoxidase staining (40×) is shown in Panels g–h. For Panels a–c, *n* = 3 WT PBS, *n* = 9 WT CFH, *n* = 3 NLRP3KO PBS, *n* = 11 NLRP3KO CFH. For Panel d, *n* = 3–4 per group

We have previously shown that intra‐tracheal CFH causes significant neutrophilic inflammation in the airspace and increases alveolar–capillary permeability after 24 hr (Shaver, Upchurch, et al., 2016). To determine the impact of NLRP3 during acute lung injury, mice lacking NLRP3 (NLRP3KO) or WT controls were treated with IT CFH. Loss of NLRP3 reduced inflammation in the airspace, with 2.7‐fold reduction in total inflammatory cell numbers (Figure 6a). There was a lack of neutrophil influx in the absence of NLRP3 (Figure 6b). Loss of NLRP3 had no effect on alveolar‐capillary barrier integrity as measured by BAL protein levels or wet‐to‐dry lung weights (Figure 6c–d). NLRP3 deletion had no effect on histologic lung injury as is shown in Figure 6e–f. Lack of NLRP3 attenuated CFH‐dependent myeloperoxidase detection (Figure 6g–h). There were no significant differences in BAL concentrations of IL‐1β, TNF‐μ, CXCL‐1, IL‐6, or IL‐10 between WT and NLRP3KO mice at this time point (data not shown).

**FIGURE 7:**
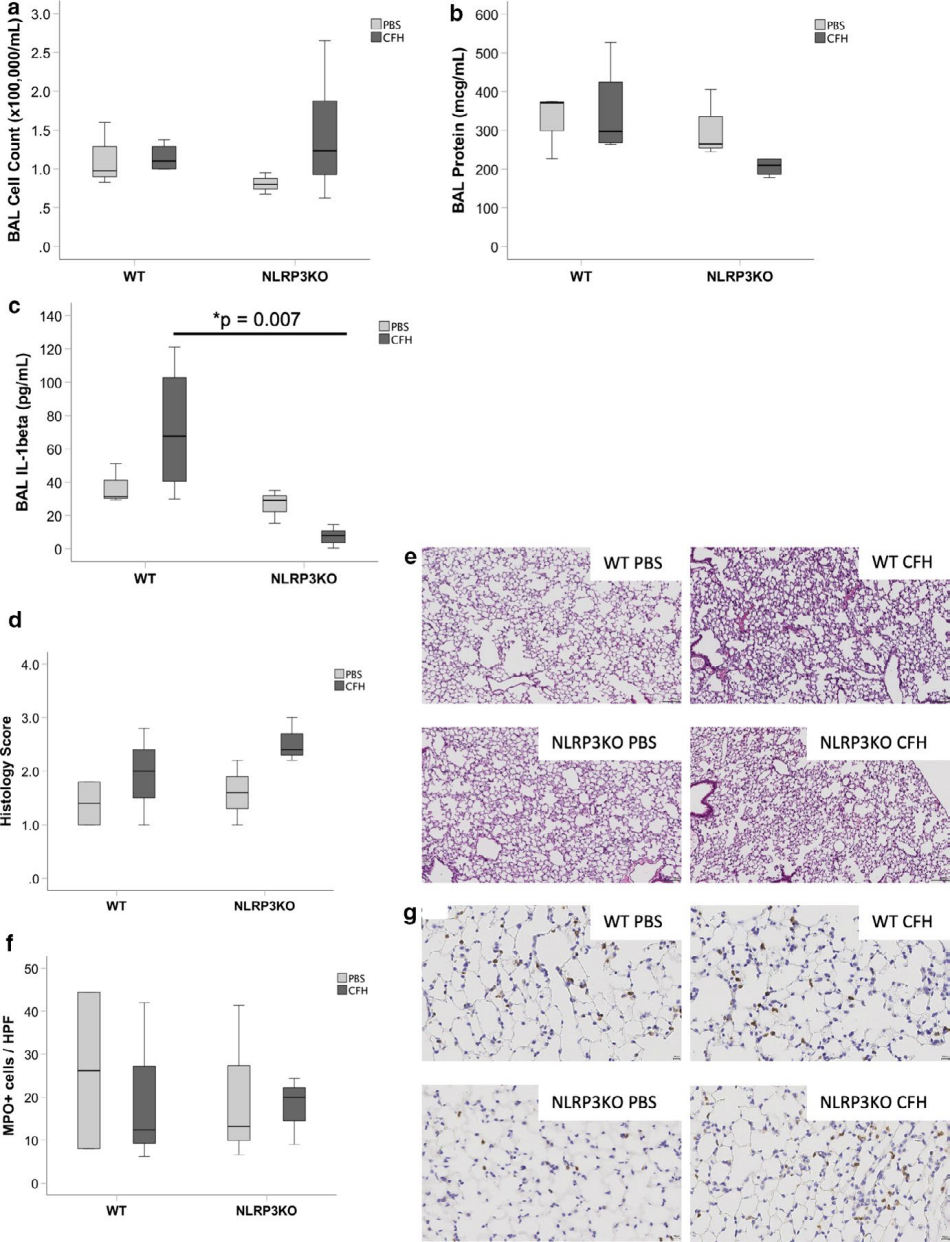
NLRP3 inflammasome has limited influence on cell‐free hemoglobin‐induced lung injury after 2 hr. NLRP3KO mice or WT controls were exposed to intra‐tracheal CFH (100 μg). There were no significant differences in (a) total BAL cell counts (*p* = .284) or (b) BAL protein (*p* = .263). (c) IL‐1β was significantly higher in WT mice after CFH compared to NLRP3KO mice (*p* = .007). There were also no NLRP3‐dependent changes in (d–e) histological lung injury (*p* = .204) or (f–g) myeloperoxidase staining (*p* = .948) in CFH‐injured lungs. Representative histology images for each treatment group are shown in Panels e (20×) and g (40×). *n* = 3 for PBS, *n* = 5 for CFH groups

Because the NLRP3 inflammasome can be assembled within a few hours, we next tested whether NLRP3 was critical for early inflammation due to CFH. Two hours after IT CFH, there were no significant differences between WT and NLRP3KO mice in total BAL cell counts or BAL protein (Figure 7a and b). NLRP3KO mice had reduced BAL concentrations of IL‐1β 2 hr after IT CFH (Figure 7c), but had no significant differences in TNF‐α, CXCL‐1, IL‐6, or IL‐10 (data not shown). Assessment of histologic lung injury showed increased injury after IT CFH in both WT and NLRP3KO mice, but there was no statistically significant difference between WT and NLRP3KO mice (Figure 7d and e). Similarly, there was no difference in the number of myeloperoxidase positive cells in the lung at this timepoint (Figure 7f and g).

### 3.5 Cell‐free hemoglobin did not induce NLRP3‐dependent cell death

**FIGURE 8:**
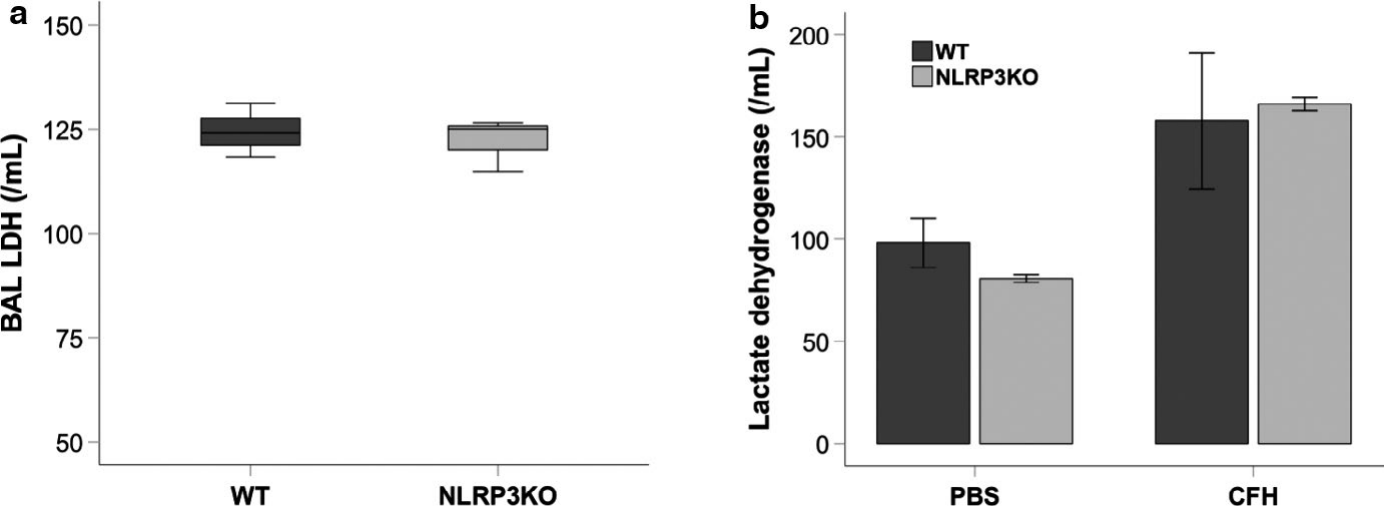
Cell‐free hemoglobin does not induce NLRP3‐dependent cell death. Release of lactate dehydrogenase (LDH) from injured cells was measured in bronchoalveolar lavage fluid from mice treated with intratracheal hemoglobin (a) or from supernatants from primary alveolar macrophages exposed to CFH ex vivo (b) demonstrated no NLRP3‐dependent induction of cell death. *n* = 3–8 per group

NLRP3 can trigger a form of inflammasome‐dependent programmed cell death that results in cell lysis and release of LDH. To test whether CFH exposure resulted in cell death, we measured LDH release in bronchoalveolar lavage fluid. There were no significant differences in BAL LDH between WT and NLRP3KO mice given intratracheal hemoglobin (Figure 8a). Similarly, there were no significant differences in LDH release from primary alveolar macrophages exposed to CFH (Figure 8b). This suggests that CFH does not induce a significant amount of NLRP3‐dependent cell death.

## 4 DISCUSSION

In this study, we identified that cell‐free hemoglobin induces the NLRP3 inflammasome in macrophages in a TLR4‐dependent manner and that NLRP3 was required for maximal recruitment of inflammatory cells into the airspace. Together, these findings provide new insight into the mechanisms through which CFH causes lung injury.

The importance of NLRP3 in acute lung injury has been well established in both human patients and in experimental models (Hosseinian et al., 2015; Lee et al., 2016; Santos, Kutuzov, & Ridge, 2012). NLRP3 gene expression is upregulated in alveolar macrophages of patients undergoing mechanical ventilation (Kuipers et al., 2012). Grailer and colleagues were first to show that NLRP3 was required for LPS‐induced acute lung injury in mice and validated their results in models of complement‐induced lung injury (Grailer et al., 2014). Other studies have demonstrated a role for NLRP3 in lung injury induced by sepsis, mechanical ventilation, (Jones et al., 2013; Kuipers et al., 2012) hyperoxia, (Fukumoto et al., 2013) burns, (Han et al., 2015) bleomycin, (Santos et al., 2015) acid aspiration, (Mizushina et al., 2019) and primary graft dysfunction after lung transplantation (Cantu et al., 2013). In addition, NLRP3 is a key regulator of infectious inflammation, particularly during bacterial and viral pneumonia (Santos et al., 2012).

The current study shows that CFH was sufficient to induce upregulation of the NLRP3 inflammasome in macrophages and that NLRP3 contributes to airspace inflammation in CFH‐induced acute lung injury. These results are consistent with the work of Nyakundi and colleagues, who demonstrated that hemoglobin contributed to NLRP3 activation after intravascular hemolysis (Nyakundi et al., 2019). Our work extends this knowledge by showing that NLRP3 in airspace macrophages is induced by CFH. In addition, our work builds on previous data showing that heme, which can be released from CFH, is capable of activating NLRP3 through Syk‐mediated pathways after TLR4 stimulation by lipopolysaccharide (Dutra et al., 2014). We demonstrate that CFH‐dependent NLRP3 induction in macrophages required TLR4, similar to previous work showing that, after TLR4 activation, heme could catalyze induction and assembly of the NLRP3 inflammasome (Dutra et al., 2014; Erdei et al., 2018). We did not specifically study whether the effect of CFH on NLRP3 required release of heme because our prior work demonstrated that heme did not induce airspace inflammation after intra‐tracheal administration of CFH (Shaver, Upchurch, et al., 2016). Our results show little impact of NLRP3 on cell‐free hemoglobin‐induced lung permeability, with no significant changes in BAL protein, histologic evidence of edema, or lung wet‐to‐dry weight ratios, suggesting that the effects of CFH on these features of lung injury may occur through mechanisms unrelated to inflammasome activation.

Further work is needed to fully understand how CFH interacts with NLRP3. Our data suggest that CFH is sufficient to induce NLRP3 and IL‐1β secretion in the absence of LPS. This may be because hemoglobin is able to directly bind TLR4 (Kwon et al., 2015). It is also possible that CFH engages additional cell surface receptors, such as RAGE, or acts through additional intermediates including HMGB1 to result in NLRP3 production. Our data also support the concept that CFH is capable of alternative activation of NLRP3 (Gaidt et al., 2016) in that CFH is sufficient to increase NLRP3. The importance of mitochondrial dysfunction (Chintagari et al., 2016) and oxidative stress (Kato, Steinberg, & Gladwin, 2017) in response to CFH may facilitate its effects on NLRP3. Furthermore, CFH appears to have differential effects on caspase‐1 and caspase‐11 through TLR4, suggesting that CFH may differentially affect canonical and non‐canonical NLRP3 signaling. Caspase‐11 can be triggered by intracellular LPS (Huang et al., 2019) whereas extracellular LPS leads to caspase‐1 activation via TLR4. It is possible that CFH could similarly have differential effects on these caspases related to its cellular location. Another possibility is that CFH may engage other signaling pathways such as Syk to augment caspase‐1 in the absence of TLR4. Further clarification is needed to determine whether CFH, TLR4, and the NLRP3 inflammasome directly interact or whether there are intermediate signaling pathways involved.

In summary, these data identify NLRP3 as an important mediator of the inflammatory responses to CFH in the airspace. Since CFH is elevated in the airspaces of the majority of patients with severe acute lung injury, (Bastarache et al., 2012) greater understanding of the mechanisms through which CFH induces acute lung injury and augments pre‐existing lung injury may lead to discovery of novel therapeutic agents.

## CONFLICT OF INTEREST

The author(s) declare no conflicts of interest.

## AUTHOR CONTRIBUTIONS

CMS and JAB conceived the study. CMS, JAB, and SRL designed the experiments. CMS, SRL, SP, FS, and NP performed the experiments and analyzed data. CMS drafted the manuscript. LBW and JAB substantively revised the manuscript. All authors approve the final manuscript.

## Data Availability

The datasets generated during and/or analyzed during the current study are available from the corresponding author on reasonable request.
